# Panbinostat decreases cFLIP and enhances killing of cancer cells by immunotoxin LMB-100 by stimulating the extrinsic apoptotic pathway

**DOI:** 10.18632/oncotarget.20263

**Published:** 2017-08-14

**Authors:** Xiu-Fen Liu, Qi Zhou, Raffit Hassan, Ira Pastan

**Affiliations:** ^1^ Laboratory of Molecular Biology, Center for Cancer Research, National Cancer Institute, National Institutes of Health, Bethesda, Maryland 20892, USA; ^2^ Thoracic and Gastrointestinal Oncology Branch, Center for Cancer Research, National Cancer Institute, National Institutes of Health, Bethesda, Maryland 20892, USA

**Keywords:** combination therapy, apoptosis, cFLIP, pancreatic cancer, mesothelin

## Abstract

LMB-100 (RG7787) is a recombinant immunotoxin, which kills mesothelin-expressing cancer cells and now being evaluated in phase 1 trials. To enhance the anti-tumor activity of LMB-100, we have searched for agents, already approved for cancer therapy, that can be combined with LMB-100 to increase its efficacy. Panbinostat is a pan-histone deacetylase inhibitor that is used to treat multiple myeloma. We incubated different types of cancer cells with panbinostat and LMB-100 and found that they interacted synergistically to cause cell death. We found that panbinostat and the combination increased levels of mRNAs encoding TNF/TNFR family members, as well as *BNIP3L* and *CASP-9*, and markedly decreased mRNA levels for *c-FLIP* and *BID*. Western blots confirmed a fall in levels of cFLIP protein and a rise in BNIP3L and caspase-9. The combination also increased levels of cleaved BID (t-BID), cleaved-capsase-3 and −8 and PARP. To assess the importance of the fall in cFLIP levels, we treated cells with the cFLIP inhibitor, Rocaglamide, and found it also enhanced killing of tumor cells by LMB-100. LMB-100, which activates the intrinsic pathway of apoptosis, and panbinostat, which activates the extrinsic pathway, work in a synergistic manner to kill cancer cell lines.

## INTRODUCTION

Histone deacetylases carry out the deacetylation of histone and non-histone proteins. They modulate gene expression through chromatin remodeling, and regulate a variety of cell functions, including cell cycle, apoptosis, cell survival, angiogenesis and metastasis. Therefore, histone deacetylases are considered promising targets for cancer therapy [1–3]. Panbinostat is a potent pan-histone deacetylase inhibitor that prolongs histone hyper-acetylation in tumor cells. Panbinostat (Farydak) was recently approved by the FDA for the treatment of multiple myeloma (http://www.myelomabeacon.com/news/2015/02/23/Farydak-panobinostat-FDA-approval). It has also shown anti-tumor activity in clinical trials in T cell lymphomas, Hodgkin lymphoma and myeloid malignancies [4, 5]. In preclinical studies with solid tumors, panbinostat has been found to suppress the growth of triple negative breast cancer, prostate cancer, hepatocellular carcinoma and other types of cancer [6–9].

Recombinant immunotoxins (RITs) are chimeric proteins that contain an antibody fragment directed against a tumor cell surface antigen attached to a protein toxin. RITs kill cells by inhibiting protein synthesis, which results in the induction of apoptosis [10]. Mesothelin is a cell surface protein that is expressed on many solid tumors including mesothelioma, and carcinomas of the pancreas, lung, ovary, stomach, breast and bile duct. Its expression on normal cells is limited to mesothelial cells and not on essential organs [11, 12]. To target mesothelin expressing cancers, we have developed a new RIT (LMB-100, previously named RG7787) in which a humanized Fab is attached to a 24 kDa fragment of *Pseudomonas* exotoxin A containing mutations in B and T cell epitopes designed to lower immunogenicity and decrease non-specific side effects [13, 14]. As a single agent LMB-100 has shown substantial anti-tumor activity in mice bearing xenografts of human cancers of the pancreas, breast, stomach and lung and mesothelioma [14, 15]. In addition, we have observed synergy and complete regressions of tumors in mice, when LMB-100 was combined with a taxane or with Dactinomycin [13, 16–18]. One special feature of RITs is that they kill cells by inhibiting protein synthesis, whereas other anti-cancer agents act by different mechanisms [10]. By treating tumors with agents with different mechanisms of action, we observed profound tumor regressions in mice. LMB-100 is now in clinical trials for the treatment of mesothelioma as a single agent and in combination with Abraxane for the treatment of pancreatic cancer (clinical trials.gov [NCI Protocol NCI-16-C-0127) [12, 19].

Our current goal is to identify other FDA approved agents that can be combined with LMB-100 for cancer treatment, because combination treatment with anti-cancer drugs is necessary to achieve the best outcome in patients. We report here that combining low doses of panbinostat with LMB-100 synergistically kills many different cancer cell lines. Mechanistic studies show that panbinostat activates apoptosis by decreasing expression of the cell death inhibitor cFLIP and increasing levels of BNIP3L and caspase-9 in multiple cell lines. Thus, activation of the extrinsic pathway by panbinostat combined with activation of the intrinsic pathway by LMB-100 leads to synergistic killing of many cancer cell lines.

## RESULTS

**Figure 1:**
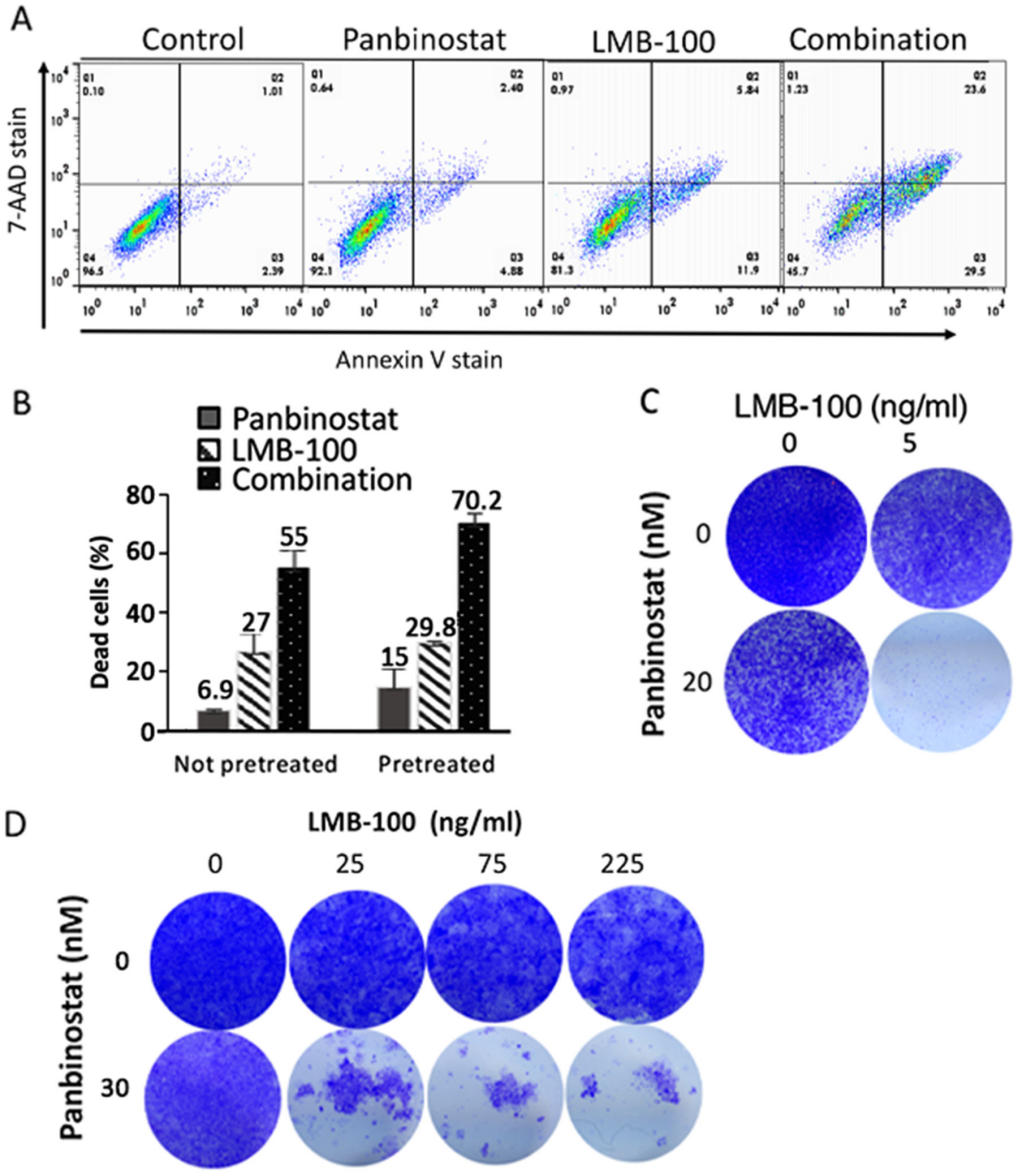
Pan combination enhanced with lMB-100 activity synergistically KLM1 cells were treated with 20 nM of panbinostat, 10 ng/ml LMB-100 or both (Combination) and incubated for 3 days. The cells were all collected and stained with 7-AAD and Annexin-V. Typical staining profile were shown **(A)**. **(B)** KLM1 cells were pretreated with 5 nM panbinostat for 6 hours (pretreated) or not pretreated, and then were treated with 20 nM panbinostst or LMB-100 5 ng/ml for 3 days. Dead cells include 7-AAD, Annexin V or both positive cells. The percentage of dead cells was calculated by subtracting the control dead cells. **(C)** and **(D)** KLM1 (C) or Panc3.014 cells (D) were treated as in (A). Dead cells were washed and fresh media were added to allow the live cells to recover. Cells were fixed and stained with crystal violet 4 days later.

To determine if panbinostat can enhance killing of tumor cells by LMB-100, we treated the pancreatic cancer cell line KLM1 with 5 ng/ml LMB-100 alone, 20 nM panbinostat alone or both for 72 hours; the cells were then stained with 7-AAD and Annexin V to measure the percentage of dead and apoptotic cells by flow cytometry. As shown in Figure 1A, treatment with panbinostat or LMB-100 alone slightly increased the staining with Annexin V alone or Annexin V plus 7-AAD, whereas combination therapy greatly increased the number of Annexin V and Annexin V plus 7-AAD positive cells. Figure 1B shows that 6.9% of the cells were dead with 20 nM panbinostat alone, 27% of the cells were dead with LMB-100 and 55% of the cells were dead with the combination. The combination Index (CI) was calculated to be 0.5 indicating the agents are acting in a synergistic manner to kill cells. To determine if pretreatment with panbinostat improves killing of KLM1 cells, we found that 20-30 nM pretreatment for 6-24 hours increased cell death by panbinostate alone, but 5 nM did not increase cell killing. Panbinostat was more effective under these conditions, increasing the number of dead cells in the combination group to 70% (P=0.046, Figure 1B)

Flow cytometry is an excellent way to determine the percentage of cells killed, but is not as useful to follow inhibition of cell growth or cell death of mass populations over time. To visualize the effects of LMB-100 and panbinostat, KLM1 cells were plated in 24-well dishes, treated for 3 days and then fresh media without drugs added and cells incubated for 4 more days to allow any living cells to recover. Figure 1C shows images of plates stained with crystal violet 7 days after plating. Single agent LMB-100 at 5 ng/ml or panbinostat at 20 nM reduced the number of KLM1 cells. However, only the combination eliminated the majority of the cells.

To investigate if panbinostat could enhance the cytotoxic activity of LMB-100 on another mesothelin expressing cancer cell, we tested the mesothelioma HAY cell line (Supplementary Figure 1A) using flow cytometry to assess the response. One group of cells was pre-treated with 5 nM panbinostat and the other was not. In both cases, the combination of panbinostat and LMB-100 synergistically killed the HAY cells (CI=0.55). We also evaluated cell killing by allowing the surviving cells time to recover after treatment as described for KLM1 cells. Supplementary Figure 1B showed that the combination killed almost all the HAY cells.

To determine if the synergy occurs on other cancer cell lines, we examined six other cell lines; pancreatic line (BxPC3), mesothelioma cell line RH16 (recently derived from a patient), two stomach cell lines (MKN28 and NUGC4), cervical cancer cells (KB31/HeLa), and a lung cancer cell line (L55). As shown in Table 1, the combination of LMB-100 and panbinostat is synergistic in killing all these cell types (CI < 1).

**Table 1:** Synergistic killing of tumor cells with panbinostat and LMB-100

Cell Lines	Tumor types	Control	Panb	LMB-100	Panb +LMB-100	Synergy score (Cl)
KLM-1	Pancreatic	6.5±1.3	28.2±6.0	36.8±0.6	79.9±3.5	0.62
BxPC3	Pancreatic	9.8±2.1	19.3±2.2	13.5±0.2	35.4±2.9	0.50
HAY	Mesothelioma	5.5±1.0	13.2±0.5	31.8±0.1	64.±1.5	0.55
RH16	Mesothelioma	10.4±0.7	10.7±0.5	15.6±0.1	35.3±2.4	0.22
MKN28	Stomach	4.9±2	15.2±1.8	5.2±0.1	57.1±6.4	0.20
NUGC4	Stomach	7.7±1.2	14.6±1.2	8.8±0.2	53.9±0.5	0.17
KB 31	Cervical	5.0±0.5	9.2±0.8	6.2±2.1	56.8±0.7	0.10
L55	Lung	9.9±0.35	19.8±1.1	9.3±2.1	35.7±8.8	0.36

Numbers are percentage of dead cells subtracted from control with SEM. Tumor cells were preincubated with panbinostat (Panb) at 5 nM for 6 hours, then incubated with either 10 nM (RH16, NUGC4), or 20 nM (KLM1, HAY, MKN28, L55), or 30 nM (BxPC3, KB 31) of panbinostat, with or without LMB-100 at 5 ng/ml (HAY, NUGC4), or 10 ng/ml (KLM1, MKN28, RH16), or 50 ng/ml (L55), or 100 ng/ml (BxPC3 and KB31) for 3 days for all cells except RH16 and MKN28, which were treated for 4 days. Cell death was determined by FACS analysis after staining of annexin-V and 7-AAD. The data was generated from 2 or 3 separate experiments. CI was calculated by the Bliss independence model {CI=(Ea+Eb-EaEb)/Eab}.

Pancreatic cancer cell lines Panc3.014 and HTB80 have low levels of the pro-apoptotic BAK protein and were previously found to be immunotoxin resistant [20]. Figure 1D shows that LMB-100 at 25, 75 or 225 ng/ml cannot kill the resistant Panc3.014 cells. However, the combination of LMB-100 with 20 nM panbinostat killed almost all the cells. Similarly, Supplementary Figure 1C shows that the combination of LMB-100 and panbinostat kills most of the HTB80 cells, whereas panbinostat alone or LMB-100 alone did not. These data show that combination therapy is effective in enhancing killing of resistant, as well as sensitive cells.

### Mechanism studies

The killing of cells by LMB-100 is complex and involves binding of LMB-100 to mesothelin on the cell membrane, internalization by endocytosis, release of the toxin from the Fab by furin, trafficking to the endoplasmic reticulum, transfer to the cytosol where elongation factor-2 (EF2) is located, ADP-ribosylation and inactivation of EF2, arrest of protein synthesis, degradation of rapidly turning over proteins like MCL-1, activation of BAK and initiation of the apoptotic cascade [10]. We examined several of these steps to determine the mechanism of synergy.

**Figure 2:**
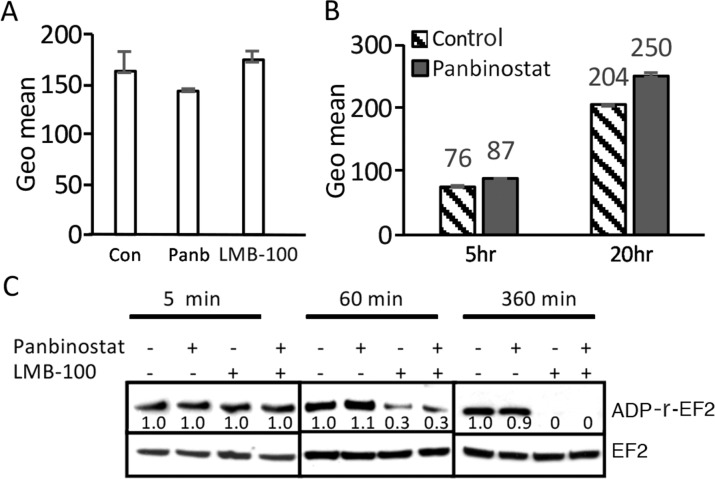
Panbinostat had minimal effect in the uptake of LMB-100, and did not affect ADP-ribosylation **(A)** Panbinostat treatment did not change surface mesothelin expression: KLM1 cells were treated with either 5 ng/ml LMB-100 or 20 nM panbinostat (Panb) for 3 days, surface expression was measured by staining with anti-mesothelin Ab and then with PE-labelled secondary Ab. Fluorescence intensity is shown as geomean. **(B)** KLM1 cells pretreated with 5 nM panbinostat for 16 hours and then exposed to10 ng/ml LMB-100-Alexa 647 with or without 20 nM panbinostat for 5 or 20 hours. The fluorescence intensity was measured by flow cytometry. **(C)** KLM1 cells were pretreated overnight with 5 nM panbinostat, then incubated with 50 ng/ml LMB-100 with or without 20 nM panbinostat at indicated times. ADP ribosylation assays were perform as described in Materials and Methods. The relative signal intensities were obtained by scanned Western blot and analyzed by NIH image.

To determine if panbinostat or LMB-100 treatment can increase surface mesothelin levels, which should result in increased LMB-100 uptake, we treated KLM1 cells with panbinostat (20 nM) or 5 ng/ml LMB-100 for 3 days and measured the amount of cell surface mesothelin with an anti-mesothelin antibody. The majority of cells will survive for 3 days at these concentrations. As shown in Figure 2A, surface mesothelin did not increase after 3 days of treatment with either panbinostat or LMB-100. We could not measure the effect of combination treatment for 3 days, because combination treatment caused death of many cells. To determine if panbinostat treatment can stimulate LMB-100 uptake, we pre-incubated KLM1 cells with 20 nM panbinostat for 16 hours and then added 10 ng/ml LMB-100 -Alex647 with or without 20 nM panbinostat for 5 or 20 hours. The data shows there is a very small increase (10 or 20%) in uptake by KLM1 cells (Figure 2B) at 5 or 16 hours, respectively. These small changes are not large enough to contribute to the large change in cell death we observed.

After internalization and furin cleavage, the active portion of the toxin is transferred to the endoplasmic reticulum and then to the cytosol, where it ADP-ribosylates EF2. To assess if ADP-ribosylation of EF2 was affected by panbinostat, we performed EF2-ribosylation assays (Figure 2C). Cell lysates from cells treated with LMB-100, panbinostat or both for various periods of time were prepared and SS1P and NAD-biotin were added to the extracts, which were then incubated for 1 hour to allow the added SS1P to catalyze the ADP-ribosylation of unmodified EF2. This assay measures how much EF2 remains unmodified by the toxin and therefore active after the cells were exposed to LMB-100. We found that the amount of EF2 available for ADP-ribosylation was not changed after cells were treated for 5 minutes. After 60 minutes the amount was decreased by 70% in both cells treated with LMB-100 alone or LMB-100 and panbinostat. After 6 hours of treatment, all the EF2 was modified and not available for modification in cell free extracts. These results show that panbinostat does not enhance the rate of modification of EF2 by the toxin and indicates panbinostat acts after the ADP-EF2-ribosylation step.

### Changes in levels of RNA for apoptotic genes

Because the primary action of panbinostat is on chromatin to inhibit histone deacetylation, and because HDAC inhibitors can affect the expression of apoptotic genes [3, 21], we examined the expression of genes in the apoptotic pathway after 16 hours of treatment with panbinostat or LMB-100 or both on KLM1 cells and MKN28 cells. The ratios of RNA levels compared with control levels are listed in Supplementary Table 1.

We observed that the levels of mRNA for housekeeping genes (*ACTN*, *GAPDH*, *B2M*, *HPRT1*) are largely unchanged, but many others are affected. Table 2 shows genes whose mRNA levels are increased or decreased over 3-fold in both cell lines by panbinostat. RNAs that are increased include TNF/TNFR family genes: *CD40* and *TNFRSF9 (CD137*) and the pro-apoptotic genes *BINP3L* and *CASP9* and *CIAP2*. In contrast *CFLAR (C-FLIP), BID, P53* and *NF-κB1* RNA are decreased 5-10-fold by panbinostat treatment. Besides genes that are commonly changed in both cell lines, panbinostat increased other TNF/TNFR family members greatly; for example, *TNFRSF1B* is increased about 20-fold only in KLM1 cells, and *TNFRSF11B* increased about 6-fold only in MKN28 cells (Supplementary Table 1). Cells treated with LMB-100 alone show increased levels of RNA for several genes in the TNF pathway and also show increases in *GAD45A* expression as previously reported [18]. Of interest, the combination treatment stimulated more than a single agent panbinostat or LMB-100, for example, *CIAP2* (*BIRC3), CASP9, CD40, and CD137.* But *TP53* and *NF-κB* changed less in the combination.

**Table 2:** Apoptotic genes regulated by panbinostat

	KLM1			MKN28		
**Panbinostat**	**LMB-100**	**Combination**	**Panbinostat**	**LMB-100**	**Combination**
BNIP3L	3.8	0.5	2.9	4.6	0.8	2.7
CASP9	3	1.4	4.9	3.3	1.5	4.9
CD40	4.2	0.8	5.1	14	0.9	21
CD137	38	14	86	56	7.8	113
CIAP2	5.5	5	17	5.3	4	12
BID	0.15	0.95	0.25	0.1	0.95	0.1
cFLIP	0.2	2.1	0.2	0.15	1.5	0.3
NFκB1	0.2	2.7	0.4	0.2	1.8	0.5
TP53	0.1	2	1.1	0.2	1	0.4

KLM1 or MKN28 cells in duplicate were pretreated with 5 nM panbinostat for 6 hours, then 20 nM panbinostat was added with or without 50 ng/ml LMB-100 for 18 hours. RNA were extracted and analyzed by apoptotic array (Qiagen). The numbers represent fold changes in RNA compared with control from an average of two samples. Genes that changed 3-fold or more by panbinostat, in both KLM1 and MKN28 cells, are presented here.

### Changes in levels of apoptotic proteins

**Figure 3:**
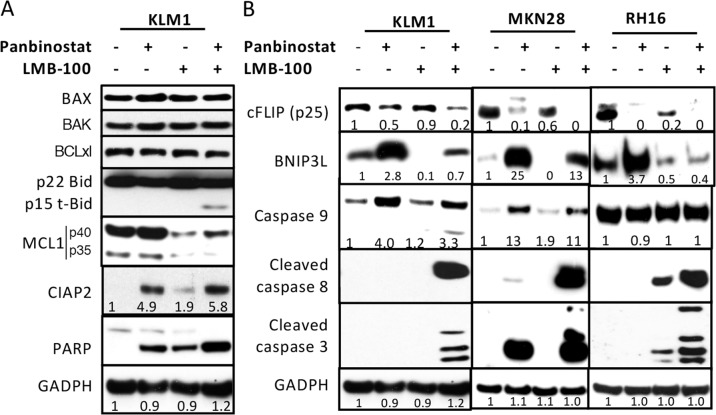
Western blot analysis of protein changes KLM1 cells were preincubated with 5 nM panbibostat for 6 hours, then 20 nM panbinostat was added with or without 100 ng/ml LMB-100 for 24 hours **(A)**. **(B)** KLM1, RH16 and MKN28 cells were similarly treated as in (A), except that RH16 cells were treated with 10 nM of panbinostat. Cell lysates were analyzed by Western blot using specific antibodies against each protein indicated. The fold changes were generated by scanning the Western blot images and the relative level analyzed by NIH Image J.

To determine if the changes in mRNA levels produced by panbinostat or LMB-100 are reflected in changes in protein levels, we carried out Western blots on selected proteins using KLM1 cells. As shown in Figure 3A and 3B, the Bcl2 family protein members BAX, BAK and BCLxl did not change with treatment. Panbinostat did cause an increase in the pro-apoptotic proteins BNIP3L and Caspase 9 and enhanced PARP cleavage. It also caused a fall in cFLIP and an increase in CIAP2. LMB-100, as expected, caused a fall in MCL-1 and in BNIP3L and enhanced PARP cleavage. The most striking changes were observed in cells treated with both agents. We observed a decrease in cFLIP, and an increase in cleaved t-BID, cleaved caspase-8, cleaved caspase-3, and cleaved PARP. These changes indicate a massive activation of the apoptotic pathways in the combination treated cells.

To study if the changes in apoptotic proteins observed in KLM1 cells occurred in other cell lines, we treated MKN28 and RH16 cells with panbinostat, LMB-100 or the combination. In MKN28 cells, panbinostat decreased cFLIP and increased BNIP3L and increased caspase-9 and the combination further decreased cFLIP and enhanced cleavage of caspase-8 and caspase-3. In RH16 cells, panbinostat lowered cFLIP and elevated BNIP3L, and the combination lowered cFLIP and BNIP3L and greatly enhanced cleavage of caspase-3 and caspase-8. Caspase-9 did not change in RH16 cells, in which the level in untreated cells is very high. Overall, the major changes in apoptotic proteins were similar in all three cancer cell lines.

### C-FLIP inhibitor Rocaglamide enhances LMB-100 activity

**Figure 4:**
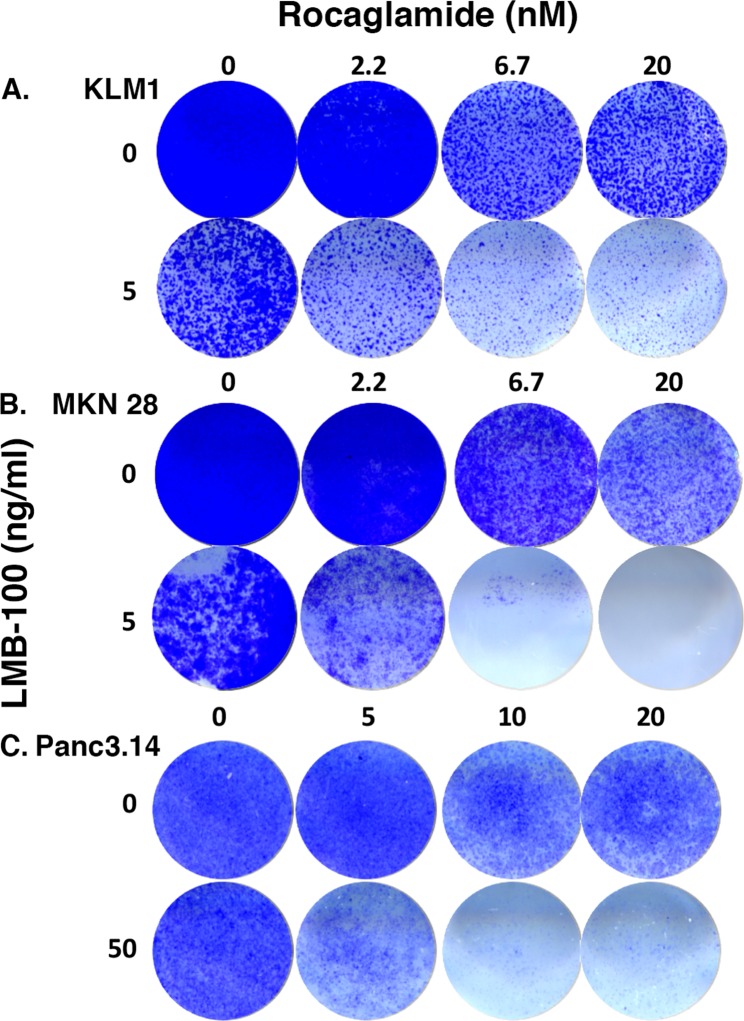
c-FLIP inhibitor increased LMB-100 cell killing KLM1 **(A)**, MKN28 **(B)** or Panc3.014 **(C)** cells were plated on 24 well plates. After overnight culture, the cells were treated with RocA at indicated concentration for 45 minutes, then LMB-100 added with or without the RocA for 3 days. The cells were then replenished with fresh media without drugs and grown for another 4 days. The surviving cells were stained with crystal violet and images were scanned.

Because c-FLIP RNA and protein changed dramatically in all three cell lines, we examined c-FLIP to see if it has an important role in enhancing LMB-100 activity by treating cells with a cFLIP inhibitor. We assessed cell killing by staining surviving cells with crystal violet. As shown in Figure 4A, when KLM1 cells were treated with 2.2 nM Rocaglamide, growth was not affected but higher concentrations decreased cell numbers in a concentration dependent manner. The addition of LMB-100 greatly enhanced cell killing. Similarly, Rocaglamide combined with LMB-100 at 5 ng/ml enhanced killing of MKN28 cells (Figure 4B) as well as resistant Panc3.014 cells, which are not killed by 50 ng/ml LMB-100 alone (Figure 4C).

## DISCUSSION

The recent FDA-approval of panbinostat for the treatment of multiple myeloma stimulated us to evaluate it as a combination therapy in solid tumors with LMB-100. We found that panbinostat enhanced the killing of a variety of mesothelin-expressing tumor cells in a synergistic manner. These include mesothelioma and cancers of the pancreas, lung, cervix and stomach.

There are many studies showing that panbinostat inhibits the growth of various cancers, and has shown strong pro-apoptotic and anti-proliferative effects in various tumor models [4, 21, 22]. The mechanism of panbinostat action has not been extensively investigated. It was shown to affect cell cycle protein p21^cip1/waf1^ and the stability of proteins involved in the canonical (death-receptor and mitochondria related) and alternative cell death pathways, e.g. ER stress [7, 21]. We report here that panbinostat increases levels of mRNA of several TNF/TNFR family members, *CD40* and *CD137* in both KLM1 and MKN28 cells. Also, *TNFR1B was increased* in KLM1cells and *TNFR11B* was increased in MKN28. Panbinostat also substantially decreased *c-FLIP* RNA and protein. The fall in the inhibitory protein cFLIP combined with elevation of the other components of the extrinsic pathway leads to extrinsic pathway activation, and when combined with activation of the intrinsic pathway by LMB-100 leads to synergistic cell killing.

BNIP3L RNA increased about 4-fold in both KLM1 and MKN28 cells and protein levels increased about 3-fold in KLM1, 25-fold in MKN28 and about 3-fold in RH16. In contrast, LMB-100 decreased this apoptotic protein in all three cell lines. The fall in BNIP3L could be due to its short half-life combined with inhibition of protein synthesis caused by LMB-100. The restoration of BNIP3L levels in combination treated cells may reflect a balance between increased synthesis and degradation (Figure 3). This restoration may be necessary for mitochondria-initiated apoptosis. It is known that BNIP3L binds to the BCL2 protein, targets mitochondria and changes the membrane potential and releases cytochrome C [23]. We have seen an increase in cleaved t-BID and cleaved caspase-9 in KLM1 cells receiving combination treatment, indicating the intrinsic pathway is indeed activated by combination therapy.

C-FLIP was shown to be transcriptionally regulated by *NF-κB1*, the tumor suppressor gene *TP53* and other transcription factors [24]. Our RNA array analysis showed that both *NF-κB1* and *TP53* RNA was decreased 10-fold in panbinostat treated cells, indicating panbinostat may lower *c-FLIP* RNA levels by suppressing these two transcription factors. We also found that the combination treatment lowered *cFLIP* RNA and cFLIP protein was significantly lower with the combination treatment than panbinostat alone in all three cell lines. It is possible that panbinostat can regulate cFLIP protein stability. It was reported that HADC inhibitors vorinostat (SAHA) and Tubacin increase Ku70 acetylation, making the Ku70/c-FLIP complex unstable, and leading to c-FLIP degradation [25]. C-FLIP plays a pivotal role in preventing procaspase activation, and the action of c-FLIP is usuually anti-apoptotic in cancer cells [26]. Our studies using the cFLIP inhibitor Rocaglamide [27] showed that it stimulated LMB-100 killing, highlighting the role of c-FLIP in the mechanism of LMB-100 synergy in killing cancer cells. Increased c-FLIP expression has been found in colorectal, cervical, and gastric cancer [24, 26]. It is also expressed in pancreatic intraepithelial neoplasms, as well as in pancreatic ductal adenocarcinoma, whereas normal pancreatic ducts do not express c-FLIP [28, 29].

Our studies provide new clues to the pro-apoptotic mechanism of panbinostat, and provide strong evidence that the combination of LMB-100 and panbinostat should be considered for the treatment of cancers expressing mesothelin.

## MATERIALS AND METHODS

### Reagents

LMB100 (RG7787, huSS1(Fab)-LR-GGS-LO10-PE24) was supplied by Roche Innovation Center (Penzberg, Germany). Panbinostat was purchased from Selleck (Houston, TX). Antibodies detecting EF2, BAX, BAK, BID, BCLxl, MCL1, Caspase- 9 and cleaved caspase-3 and −8, PARP, CIAP2, c-FLIP, BNIP3L and GAPDH were purchased from Cell Signaling (Danvers, MA).

### Cell cultures

Human cell lines KLM1 and BxPC3 [13], MKN28 [15], KB31 [30] and HAY [18] were described previously. L55 (NSCLC) was provided by Steven M. Albelda (University of Pennsylvania, Philadelphia, PA). Human mesothelioma cell RH16 were obtained from Dr. Raffit Hassan (National Cancer Institute). All cells were grown in RPMI and supplemented with 10% fetal bovine serum and 1% penicillin-streptomycin at 37^°^C. Their identities were confirmed by short tandem repeat analysis.

### Western blot analysis

Cells were washed in PBS and lysed for 30 minutes on ice in modified RIPA buffer (50 mM Tris HCl, 150 mM NaCl, 5 mM EDTA, 1% NP40, 5 μg/ml leupeptin, 5 μg/ml aprotinin, 10 μM PMSF). After high-speed centrifugation, supernatants were analyzed by SDS-PAGE, transferred to PVDF membranes, and subjected to Western blot analysis.

### Flow cytometry analysis

Analysis of apoptotic cells: Culture cells in 6 well plates were treated with LMB-100 in combination with panbinostat or vehicle for 72 hours. Cells were harvested and stained with Annexin V-PE and 7-AAD according to instructions (BD Biosciences, San Jose, CA). Cells were analyzed on a FACS Calibur (Becton Dickinson, Franklin Lakes, NJ) and data analyzed in FlowJo (Tree Star Inc, San Carlos, CA).

Internalization of LMB-100 was measured as described [30]. Briefly, KLM1 cells were treated with or without 5 nM panbinostat overnight in 6-well plates, 10 ng/ml of LMB-100-Alexa647 with or without 20 nM panbinostat was added and cells were incubated at 37^°^C for the indicated times. Cells were washed with PBS, stripped 5 minutes with 0.2 M Glycine-HCl (pH2.5) to remove surface bound LMB-100, washed with FACS buffer (PBS with 5% FBS, 0.1% NaN_3_) and analyzed by flow cytometry using a FACS Calibur.

### ADP-ribosylation of eukaryotic EF2 (eEF2)

Cells were lysed in modified RIPA buffer; 5 μg protein from the cell lysates was incubated with 50 ng LMB-100 in ADP-ribosylation buffer (20 mM Tris-HCl (pH 7.4), 1 mM EDTA, 50 mM DTT) with 0.3 μM of 6-biotin-17-NAD (Trevigen, Gaithersburg, MD) for 60 minutes at 25°C. Samples were subjected to SDS-PAGE, followed by Western blotting with HRP-conjugated streptavidin to detect biotin-ADP-ribose-eEF2.

## SUPPLEMENTARY MATERIALS FIGURES AND TABLES





## Abbreviations

CI: combination index
EF2: elongation factor 2
eEF2: eukaryotic EF2
rITs: recombinant immunotoxins

## References

[1] Johnstone RW (2002). Histone-deacetylase inhibitors: novel drugs for the treatment of cancer. Nat Rev Drug Discov.

[2] West AC, Johnstone RW (2014). New and emerging HDAC inhibitors for cancer treatment. J Clin Invest.

[3] Manal M, Chandrasekar MJ, Gomathi Priya J, Nanjan MJ (2016). Inhibitors of histone deacetylase as antitumor agents: a critical review. Bioorg Chem.

[4] Prince HM, Bishton MJ, Johnstone RW (2009). Panobinostat (LBH589): a potent pan-deacetylase inhibitor with promising activity against hematologic and solid tumors. Future Oncol.

[5] Imai Y, Maru Y, Tanaka J (2016). Action mechanisms of histone deacetylase inhibitors in the treatment of hematological malignancies. Cancer Sci.

[6] Tate CR, Rhodes LV, Segar HC, Driver JL, Pounder FN, Burow ME, Collins-Burow BM (2012). Targeting triple-negative breast cancer cells with the histone deacetylase inhibitor panobinostat. Breast Cancer Res.

[7] Gahr S, Wissniowski T, Deike S, Strobel D, Pustowka A, Ocker M (2012). Combination of the deacetylase inhibitor panobinostat and the multi-kinase inhibitor sorafenib for the treatment of metastatic hepatocellular carcinoma - review of the underlying molecular mechanisms and first case report. J Cancer.

[8] Zopf S, Ocker M, Neureiter D, Alinger B, Gahr S, Neurath MF, Di Fazio P (2012). Inhibition of DNA methyltransferase activity and expression by treatment with the pan-deacetylase inhibitor panobinostat in hepatocellular carcinoma cell lines. BMC Cancer.

[9] Grasso C (2015). Functionally defined therapeutic targets in diffuse intrinsic pontine glioma. Nat Med.

[10] Weldon JE, Pastan I (2011). A guide to taming a toxin-recombinant immunotoxins constructed from Pseudomonas exotoxin A for the treatment of cancer. FEBS J.

[11] Pastan I, Hassan R, Fitzgerald DJ, Kreitman RJ (2006). Immunotoxin therapy of cancer. Nat Rev.

[12] Hassan R, Thomas A, Alewine C, Le DT Jaffee EM, Pastan I (2016). Mesothelin immunotherapy for cancer: ready for prime time?. J Clin Oncol.

[13] Hollevoet K, Mason-Osann E, Liu XF, Imhof-Jung S, Niederfellner G, Pastan I (2014). *In vitro* and *in vivo* activity of the low-immunogenic antimesothelin immunotoxin RG7787 in pancreatic cancer. Mol Cancer Ther.

[14] Bauss F, Lechmann M, Krippendorff BF, Staack R, Herting F, Festag M, Imhof-Jung S, Hesse F, Pompiati M, Kollmorgen G, da Silva Mateus Seidl R, Bossenmaier B, Lau W (2016). Characterization of a re-engineered, mesothelin-targeted Pseudomonas exotoxin fusion protein for lung cancer therapy. Mol Oncol.

[15] Alewine C, Xiang L, Yamori T, Niederfellner G, Bosslet K, Pastan I (2014). Efficacy of RG7787, a next-generation mesothelin-targeted immunotoxin, against triple-negative breast and gastric cancers. Mol Cancer Ther.

[16] Kolyvas E, Rudloff M, Poruchynsky M, Landsman R, Hollevoet K, Venzon D, Alewine C (2017). Mesothelin-targeted immunotoxin RG7787 has synergistic anti-tumor activity when combined with taxanes. Oncotarget.

[17] Zhang J, Khanna S, Jiang Q, Alewine C, Miettinen M, Pastan I, Hassan R (2017). Efficacy of anti-mesothelin immunotoxin RG7787 plus nab-paclitaxel against mesothelioma patient-derived xenografts and mesothelin as a biomarker of tumor response. Clin Cancer Res.

[18] Liu XF, Xiang L, Zhou Q, Carralot JP, Prunotto M, Niederfellner G, Pastan I (2016). Actinomycin D enhances killing of cancer cells by immunotoxin RG7787 through activation of the extrinsic pathway of apoptosis. Proc Natl Acad Sci U S A.

[19] Alewine C, Pastan I (2016). New life for immunotoxin cancer therapy. Clin Cancer Res.

[20] Du X, Xiang L, Mackall C, Pastan I (2011). Killing of resistant cancer cells with low Bak by a combination of an anti-mesothelin immunotoxin and a TRAIL receptor 2 agonist antibody. Clin Cancer Res.

[21] Atadja P (2009). Development of the pan-DAC inhibitor panobinostat (LBH589): successes and challenges. Cancer Lett.

[22] Di Fazio P, Schneider-Stock R, Neureiter D, Okamoto K, Wissniowski T, Gahr S, Quint K, Meissnitzer M, Alinger B, Montalbano R, Sass G, Hohenstein B, Hahn EG, Ocker M (2010). The pan-deacetylase inhibitor panobinostat inhibits growth of hepatocellular carcinoma models by alternative pathways of apoptosis. Cell Oncol.

[23] Chinnadurai G, Vijayalingam S, Gibson SB (2008). BNIP3 subfamily BH3-only proteins: mitochondrial stress sensors in normal and pathological functions. Oncogene.

[24] Bagnoli M, Canevari S, Mezzanzanica D (2010). Cellular FLICE-inhibitory protein (c-FLIP) signaling: a key regulator of receptor-mediated apoptosis in physiologic context and in cancer. Int J Biochem Cell Bio.

[25] Kerr E, Holohan C, McLaughlin KM, Majkut J, Dolan S, Redmond K, Riley J, McLaughlin K, Stasik I, Crudden M, Van Schaeybroeck S, Fenning C, O’Connor R (2012). Identification of an acetylation-dependent Ku/FLIP complex that regulate FLIP expression and HDAC inhibitor-induced apoptotsis. Cell Death Differ.

[26] Safa AR (2012). c-FLIP, a master anti-apoptotic regulator. Exp Oncol.

[27] Luan Z, He Y, He F, Chn Z (2015). Rocaglamide overcomes tumor necrosis factor-related apoptosis-inducing ligand resistance in hepatocellular carcinoma cells by attenuating the inhibition of caspase-8 through cellular FLICE-like-inhibitory protein downregulation. Mol Med Rep.

[28] Niedergethmann M, Alves F, Neff JK, Heidrich B, Aramin N, Li L, Pilarsky C, Grützmann R, Allgayer H, Post S, Gretza N (2007). Gene expression profiling of liver metastases and tumour invasion in pancreatic cancer using an orthotopic SCID mouse model. Br J Cancer.

[29] Haag C, Stadel D, Zhou S, Bachem MG, Möller P, Debatin KM, Fulda S (2011). Identification of c-FLIPL and c-FLIPS as critical regulator of death receptor-induced apoptosis in pancreatic cancer cells. Gut.

[30] Liu XF, FitzGerald DJ, Pastan I (2013). The insulin receptor negatively regulates the action of Pseudomonas toxin-based immunotoxins and native Pseudomonas toxin. Cancer Res.

