# Supplementary material for: Panbinostat decreases cFLIP and enhances killing of cancer cells by immunotoxin LMB-100 by stimulating the extrinsic apoptotic pathway
Source: Oncotarget. 2017 Aug 14;8(50):87307–16. doi: 10.18632/oncotarget.20263 (PMC5675634; doi:10.18632/oncotarget.20263)
Supplement: Supplementary file 1 [file oncotarget-08-87307-s001.pdf]

Panbinostat decreases cFLIP and enhances killing of cancer cells by immunotoxin LMB-100 by stimulating the extrinsic apoptotic pathway

SUPPLEMENTARY MATERIALS

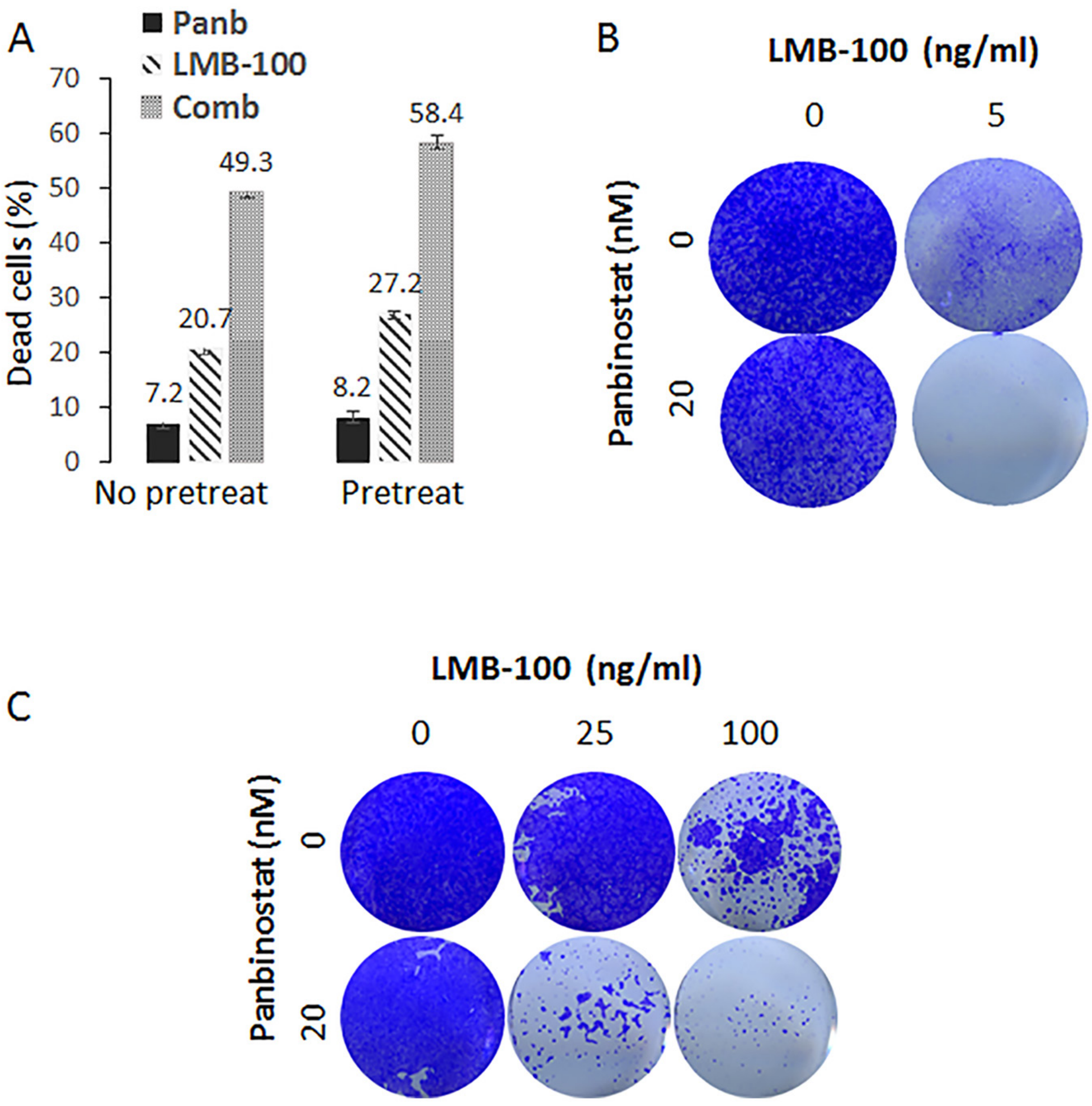

Supplementary Figure 1: Combination of panbinostat increased LMB-100 activity. (A) HAY cells were pretreated or not-pretreated with 5 nM panbinostat (panb) for 6 hours, then 20 nM panbinostat W/O 5 ng/ml LMB-100 were added for 3 days. All cells were collected and stained with annexin-V and 7-AAD before flow cytometry analysis. The graphed dead cells (%) were subtracted from control dead cells. **(B)** HAY cells were treated as in (A). The cells were washed and fresh media were added to allow the live cells to recover. Four days later cells were fixed and stained with crystal violet. **(C)** HTB80 cells were pretreated with 10 nM panbinostat overnight, then treated with 20 nM panbinostat and indicated concentrations of LMB-100 for 3 days. The surviving cells were stained with crystal violet as in B. Comb = combination.

Supplementary Table 1: Apoptotic RNA array

See Supplementary File 1
