# Supplementary material for: Panbinostat decreases cFLIP and enhances killing of cancer cells by immunotoxin LMB-100 by stimulating the extrinsic apoptotic pathway
Source: Oncotarget. 2017 Aug 14;8(50):87307–16. doi: 10.18632/oncotarget.20263 (PMC5675634; doi:10.18632/oncotarget.20263)
Supplement: Supplementary file 2 [file oncotarget-08-87307-s002.docx]

**Supplementary Table 1: Apoptotic RNA array**

| CD40LG | 3.5 | 2.5 | 1.6 | 2.4 | 4.4 | 9.8 | 1.4 | 1.4 | 1.5 | 1.0 | 1.4 | 2.2 |
| --- | --- | --- | --- | --- | --- | --- | --- | --- | --- | --- | --- | --- |
| CD70 | 1.8 | 1.7 | 1.4 | 1.3 | 2.6 | 2.5 | 4.4 | 3.7 | 0.7 | 1.7 | 8.2 | 12.2 |
| CFLAR | 0.2 | 0.2 | 2.2 | 2.1 | 0.2 | 0.2 | 0.1 | 0.2 | 1.5 | 1.6 | 0.3 | 0.3 |
| CIDEA | 3.0 | 1.4 | 0.8 | 0.8 | 1.3 | 2.3 | 1.4 | 1.4 | 1.5 | 1.0 | 1.4 | 1.6 |
| CIDEB | 1.3 | 1.3 | 0.8 | 1.0 | 1.4 | 1.7 | 2.0 | 2.3 | 1.3 | 1.2 | 2.4 | 2.8 |
| CRADD | 0.6 | 0.4 | 1.3 | 1.6 | 0.5 | 0.5 | 0.5 | 0.5 | 1.5 | 1.7 | 0.6 | 0.7 |
| CYCS | 1.8 | 1.6 | 0.9 | 1.1 | 1.6 | 2.2 | 1.4 | 1.7 | 1.0 | 0.9 | 1.8 | 2.0 |
| DAPK1 | 1.1 | 1.2 | 0.9 | 1.1 | 1.2 | 1.4 | 1.2 | 1.3 | 1.9 | 2.0 | 2.4 | 2.2 |
| DFFA | 1.5 | 1.4 | 1.0 | 1.0 | 1.7 | 1.8 | 1.5 | 1.8 | 1.6 | 1.3 | 2.1 | 2.3 |
| DIABLO | 1.7 | 1.6 | 1.4 | 1.4 | 2.6 | 2.6 | 1.4 | 1.6 | 1.4 | 1.3 | 2.0 | 2.1 |
| FADD | 0.8 | 0.8 | 1.3 | 1.3 | 0.8 | 1.0 | 0.6 | 0.6 | 1.1 | 1.3 | 0.9 | 1.0 |
| FAS | 0.3 | 0.3 | 1.3 | 1.3 | 0.3 | 0.3 | 0.8 | 0.8 | 1.1 | 1.2 | 0.8 | 0.9 |
| FASLG | 1.8 | 2.3 | 1.3 | 1.8 | 1.3 | 3.7 | 1.4 | 1.4 | 1.5 | 1.0 | 1.4 | 1.6 |
| GADD45A | 1.5 | 1.3 | 22.2 | 16.8 | 11.1 | 9.2 | 2.5 | 3.2 | 9.9 | 8.6 | 13.5 | 18.3 |
| HRK | 0.4 | 0.4 | 0.7 | 0.7 | 0.4 | 0.4 | 1.0 | 1.1 | 1.8 | 1.3 | 1.1 | 1.4 |
| IGF1R | 0.6 | 0.7 | 1.1 | 1.4 | 0.5 | 0.6 | 0.7 | 0.7 | 1.4 | 1.1 | 0.8 | 0.8 |
| IL10 | 1.6 | 1.7 | 0.8 | 0.8 | 2.0 | 2.2 | 1.7 | 8.6 | 0.9 | 1.0 | 2.9 | 10.8 |
| LTA | 4.0 | 4.1 | 2.8 | 3.7 | 5.4 | 10.7 | 1.3 | 9.6 | 1.0 | 3.2 | 4.3 | 20.8 |
| LTBR | 0.3 | 0.3 | 0.9 | 1.1 | 0.2 | 0.2 | 0.4 | 0.5 | 1.8 | 1.3 | 0.4 | 0.5 |
| MCL1 | 1.2 | 1.2 | 1.4 | 1.3 | 2.5 | 2.4 | 1.7 | 2.1 | 1.6 | 1.8 | 2.6 | 3.7 |
| NAIP | 1.3 | 1.3 | 1.0 | 1.0 | 1.3 | 1.2 | 0.9 | 0.9 | 1.2 | 1.0 | 0.9 | 1.1 |
| NFKB1 | 0.2 | 0.2 | 2.6 | 2.7 | 0.4 | 0.4 | 0.2 | 0.2 | 2.0 | 1.6 | 0.4 | 0.5 |
| NOD1 | 0.5 | 0.4 | 0.5 | 0.6 | 0.2 | 0.3 | 0.4 | 0.5 | 1.1 | 1.0 | 0.4 | 0.6 |
| NOL3 | 0.3 | 0.3 | 0.8 | 1.0 | 0.2 | 0.2 | 0.7 | 0.7 | 1.3 | 1.1 | 0.5 | 0.6 |
| PYCARD | 0.2 | 0.2 | 1.0 | 0.9 | 0.4 | 0.3 | 0.5 | 0.6 | 0.9 | 1.0 | 0.6 | 0.5 |
| RIPK2 | 0.8 | 0.8 | 1.6 | 1.6 | 1.2 | 1.1 | 1.1 | 1.2 | 1.3 | 1.3 | 1.5 | 1.3 |
| TNF | 1.1 | 1.4 | 8.9 | 7.4 | 3.7 | 2.5 | 2.0 | 8.6 | 20.1 | 16.4 | 12.5 | 14.7 |
| TNFRSF10A | 0.3 | 0.3 | 1.3 | 1.4 | 0.5 | 0.6 | 0.6 | 0.5 | 1.7 | 1.6 | 0.8 | 1.0 |
| TNFRSF10B | 1.2 | 1.0 | 2.2 | 2.1 | 1.3 | 1.5 | 2.3 | 2.2 | 1.7 | 1.5 | 2.4 | 2.3 |
| TNFRSF11B | 0.4 | 0.6 | 0.6 | 0.5 | 0.9 | 0.6 | 6.4 | 5.8 | 1.5 | 0.8 | 7.4 | 6.9 |
| TNFRSF1A | 0.2 | 0.2 | 0.9 | 1.1 | 0.3 | 0.3 | 0.6 | 0.5 | 1.3 | 1.1 | 0.6 | 0.6 |
| TNFRSF1B | 19.7 | 19.6 | 2.5 | 2.5 | 19.2 | 30.6 | 1.4 | 1.4 | 1.5 | 1.0 | 1.4 | 1.6 |
| TNFRSF21 | 0.2 | 0.2 | 0.8 | 0.8 | 0.3 | 0.3 | 0.9 | 1.0 | 1.0 | 1.2 | 1.1 | 1.2 |
| TNFRSF25 | 0.7 | 0.7 | 0.5 | 0.8 | 0.4 | 0.5 | 2.7 | 2.6 | 1.6 | 0.9 | 3.0 | 3.2 |
| TNFRSF9 | 42.5 | 34.3 | 13.2 | 15.0 | 74.1 | 89.5 | 50.4 | 62.2 | 8.0 | 6.6 | 105.4 | 121.6 |
| TNFSF10 | 0.2 | 0.2 | 0.3 | 0.4 | 0.2 | 0.3 | 0.7 | 1.5 | 0.5 | 0.9 | 0.8 | 0.9 |
| TNFSF8 | 1.2 | 1.0 | 2.7 | 2.1 | 1.9 | 2.1 | 2.3 | 0.9 | 2.8 | 4.5 | 2.1 | 0.7 |
| TP53 | 0.1 | 0.1 | 1.8 | 2.2 | 1.0 | 1.1 | 0.2 | 0.2 | 1.1 | 0.8 | 0.4 | 0.4 |
| TP53BP2 | 1.5 | 1.4 | 2.3 | 2.1 | 2.3 | 2.2 | 1.3 | 1.3 | 1.3 | 1.3 | 1.9 | 2.2 |
| TP73 | 1.9 | 1.7 | 1.4 | 1.8 | 2.2 | 4.4 | 1.0 | 0.9 | 1.8 | 2.1 | 1.5 | 2.2 |
| TRADD | 1.2 | 1.1 | 0.8 | 1.1 | 1.1 | 1.8 | 1.4 | 0.9 | 1.2 | 0.4 | 1.6 | 1.5 |
| TRAF2 | 0.9 | 0.9 | 1.3 | 1.6 | 0.9 | 1.3 | 0.7 | 0.6 | 2.1 | 1.4 | 1.8 | 1.5 |
| TRAF3 | 0.4 | 0.4 | 1.5 | 1.6 | 0.5 | 0.5 | 0.5 | 0.5 | 1.5 | 1.1 | 0.6 | 0.6 |
| XIAP | 0.7 | 0.6 | 1.1 | 1.1 | 0.4 | 0.4 | 0.7 | 0.7 | 1.2 | 1.3 | 0.7 | 0.7 |
| ACTB | 1.3 | 1.3 | 1.1 | 1.2 | 1.7 | 1.9 | 0.9 | 1.1 | 1.1 | 1.2 | 1.1 | 1.1 |
| B2M | 1.5 | 1.4 | 1.1 | 1.1 | 1.5 | 1.4 | 1.8 | 1.8 | 1.0 | 0.8 | 1.7 | 1.4 |
| GAPDH | 0.9 | 0.9 | 0.9 | 0.9 | 0.7 | 0.8 | 0.9 | 1.0 | 1.0 | 1.3 | 0.8 | 1.2 |
| HPRT1 | 0.6 | 0.5 | 1.0 | 0.9 | 0.6 | 0.6 | 0.6 | 0.4 | 1.0 | 0.9 | 0.7 | 0.6 |
| RPLP0 | 1.0 | 1.1 | 1.0 | 1.0 | 0.9 | 0.8 | 1.1 | 1.1 | 0.8 | 0.9 | 1.0 | 1.0 |

KLM1 or MKN28 cells in duplicate were treated with 20 nM panbinostat (panb) or 50 ng/ml LMB-100 (LMB) for 16 hours. The total RNA were extracted and analyzed by apoptotic array as described in Materials and Methods. Fold changes were calculated by setting untreated controls as 1, and using *ACTB, B2M, GAPDH, HPRT1 and RPLP0* as internal control using web-based data analysis (Qiagen). Comb = combination. The RNA array list represents the complete set of raw data.
